# Absence of plastidic carbonic anhydrases affects plant growth but not photosynthesis

**DOI:** 10.1093/plphys/kiag084

**Published:** 2026-02-24

**Authors:** Anna Moseler

**Affiliations:** Assistant Features Editor, Plant Physiology, American Society of Plant Biologist, Rockville, MD 20855, United States; INRES-Chemical Signalling, University of Bonn, Bonn 53117, Germany

Carbonic anhydrases (CA) are present in archaea, bacteria, and eukaryotes and catalyze the interconversion of CO_2_ and H_2_O with bicarbonate (HCO_3_^−^) and protons (Banerjee and Deshpande 2016). In cyanobacteria and C4 plants, CAs are part of the CO_2_-concentrating mechanisms, where local levels of CO_2_ are concentrated to enhance CO_2_ assimilation by Rubisco and consequently to decrease photorespiration (Cannon et al. 2010; Ludwig 2016). In C3 plants, such as Arabidopsis and most crop plants, CAs are highly abundant in mesophyll cells and it has been suggested that CAs enhance CO_2_ uptake by similarly increasing the concentration of CO_2_ (Badger and Price 1994). However, knockout of the two plastidic CAs in tobacco, βCA1 and βCA5, revealed that loss-of-function of these enzymes had no impact on CO_2_ fixation rates or photosystem II (PSII) efficiency. Instead, the respective double mutant showed a decreased free fatty acid pool and a difference in the fatty acid composition. These results lead to the assumption that in C3 plastids, stromal CAs maintain HCO_3_^−^ at equilibrium concentration to provide HCO_3_^−^ for consumption by bicarbonate-dependent carboxylation pathways (Hines et al. 2021). Further evidence for the role of CAs for bicarbonate-dependent carboxylation reactions was shown shortly after in Arabidopsis, where the stunted growth phenotype of the *βca5* mutant was partially rescued by growth on malonate, which bypasses the first step of fatty acid biosynthesis via acetyl-CoA carboxylase (Weerasooriya et al. 2022). However, it has also been shown that the *βca5* mutant had a lower chlorophyll content and exhibited lower photosynthetic performance compared with wild type (WT) and the *βca1* mutant (Sharma et al. 2023), although detailed analysis on the impact on CO_2_ fixation was not performed.

In this study published in *Plant Physiology*, Weerasooriya et al. (2026) demonstrate that the plastidic carbonic anhydrases βCA1 and βCA5 are not important for CO_2_ assimilation in Arabidopsis but that βCA5 is indispensable for plant growth under ambient CO_2_ levels.

First, the authors analyzed the growth of *βca1* and *βca5* null mutants and the respective double mutant compared to WT under different CO_2_ levels. *βca1* plants grow normally under ambient and elevated CO_2_ levels. In contrast, although the growth of *βca5* or the *βca1βca5* double mutant plants was similar to WT when grown at highly elevated CO_2_ levels, these mutants showed a severely stunted growth phenotype when grown under ambient CO_2_ levels. Continuous growth under ambient CO_2_ levels results in lethality of the mutants indicating that the presence of βCA5 is essential for plant growth.

Because the *βca1βca5* double mutant shows a similar phenotype to the *βca5* mutant, it suggests that βCA1 is not able to compensate for the loss of βCA5. However, complementation of the double mutant with either *βCA1* or *βCA5* driven by a UBIQUITIN 1 promotor rescued the growth phenotype, suggesting that it is the expression pattern of βCA1 and not its biochemical activity that fails to complement the loss of βCA5. The authors showed that *βCA1* naturally exhibits leaf-specific expression (Weerasooriya et al. 2022), but under the UBIQUITIN 1 promoter it is also expressed in the root, similar to *βCA5*, and can thus rescue the phenotype of the double mutant. These results indicate that the absence of βCA activity in roots leads to the deficiency in growth at ambient CO_2_ levels.

Because the role of plastidic βCAs was initially associated with photosynthetic CO_2_ fixation, Weerasooriya and colleagues measured next the CO_2_ assimilation rate, intercellular CO_2_ concentration, and PSII efficiency. Under highly elevated CO_2_ levels, no differences were observed between the mutants and WT. Also when the mutants were grown at the lowest CO_2_ levels where the growth was similar to WT, which was 12,000 µL L^−1^ CO_2_, no differences were observed between mutants and WT, indicating that the absence of plastidic βCAs does not impact the capacity of photosynthetic CO_2_ assimilation (Fig. 1).

**Figure 1:**
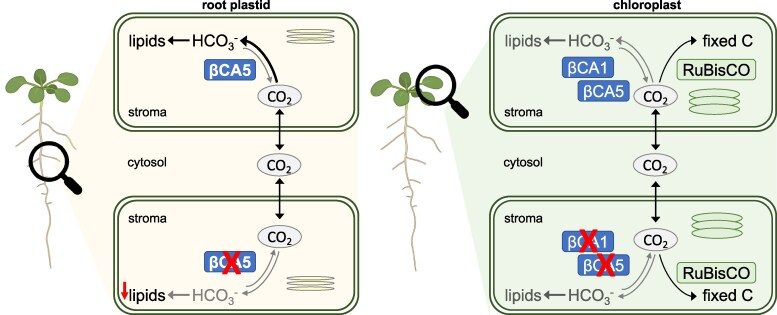
Scheme illustrating the importance of βCA5 in root plastids. CO_2_ enters the stroma via passive or facilitated diffusion across the plastidic envelope membranes. In root plastids, βCA5 catalyzes the conversion of CO_2_ to HCO_3_^–^ providing bicarbonate for lipid biosynthesis. Although βCA5 is, similar to βCA1, also expressed in leaf cells, absence of the CAs in chloroplasts have no impact on fatty acid synthesis or CO_2_ fixation.

Recently, it was shown that the deficient growth phenotype of the *βca5* mutant can be rescued by expressing LCIA (low CO_2_-inducible protein A), a bicarbonate transporter of *Chlamydomonas reinhardtii* that allows HCO_3_^–^ to enter the plastid, thus potentially increasing HCO_3_^-^ levels at the site where it is needed putatively for fatty acid biosynthesis (Förster et al. 2023). Expression of LCIA partially rescued the growth phenotype of the *βca5* mutant and the *βca1βca5* double mutant, suggesting that the supply of HCO_3_^–^ in the plastid is the growth-limiting factor. With the partial rescue of the growth phenotype, the authors were able to grow the plants under CO_2_ levels that were closer to ambient CO_2_ levels and to conduct CO_2_ assimilation rates. When grown at 1,200 μL L^−1^ CO_2_, no differences were observed between the WT and the mutants in the CO_2_ assimilation rate, intercellular CO_2_ concentration, or photosynthetic performance. This result indicates that in Arabidopsis, the plastidic CAs are not involved in enhancing CO_2_ fixation at either elevated or more ambient CO_2_ levels.

In summary, Weerasooriya et al. showed that plastidic βCAs, especially βCA5, are important for C3 plant growth but not due to an enhancement of CO_2_ fixation. This study supports previous research showing that plastidic βCAs play a role in the supply of carboxylases with HCO_3_^–^ rather than being required for photosynthetic performance (Hines et al. 2021; Weerasooriya et al. 2022). Different expression patterns and the absence of an enhanced phenotype in the *βca1βca5* double mutant suggest non-overlapping roles of both βCAs and leaves the exact physiological role of βCA1 still open. Noteworthy, βCA1 is solely present in dicots, pointing to a putatively less general function (DiMario et al. 2017). Understanding the precise role of plastidic βCAs can further help to engineer CO_2_-concentrating mechanisms into C3 crops to obtain greater Rubisco efficiency and photorespiratory bypass to increase crop productivity.

## Recent related articles in *Plant Physiology*:

- Weerasooriya et al. (2022) showed that Arabidopsis βCA5 is important for plant growth and required for HCO_3_^-^ supply for anaplerotic pathways.
- Rajarathinam et al. (2025) established a minimally invasive methodology for in vivo Rubisco activity measurement.
- Kaste et al. (2024) and Fei et al. (2025) modeled and discussed the feasibility of enhancing C3 crop yield by endowing them with a pyrenoid-based CO_2_ concentrating mechanism.

## Data Availability

No new data included in this article.
